# Positive association between weight-adjusted-waist index and dementia in the Chinese population with hypertension: a cross-sectional study

**DOI:** 10.1186/s12888-023-05027-w

**Published:** 2023-07-19

**Authors:** Wei Zhou, Yanyou Xie, Lingling Yu, Chao Yu, Huihui Bao, Xiaoshu Cheng

**Affiliations:** 1 Center for Prevention and Treatment of Cardiovascular Diseases, the Second Affiliated Hospital of Nanchang University, Nanchang, 330006 Jiangxi China; 2 Jiangxi Sub-Center of National Clinical Research Center for Cardiovascular Diseases, Nanchang, 330006 Jiangxi China; 3 Jiangxi Provincial Cardiovascular Disease Clinical Medical Research Center, Nanchang, 330006 Jiangxi China; 4 Department of Cardiovascular Medicine, the Second Affiliated Hospital of Nanchang University, Nanchang, 330006 Jiangxi China; 5 Department of Rehabilitation, The Second Affiliated Hospital of Nanchang University, Nanchang, 330006 Jiangxi China

**Keywords:** Weight-adjusted waist index, MMSE, Dementia, Hypertension

## Abstract

**Purpose:**

The links between obesity and dementia remain equivocal. Therefore, this study aimed to explore the association between weight-adjusted waist index (WWI), a new anthropometric indicator reflecting obesity, and dementia in the Chinese population with hypertension.

**Methods:**

A total of 10,289 participants with hypertension were enrolled in this cross-sectional study, a subset of the China H-type hypertension registry study. WWI was calculated as waist circumference (WC) divided by the square root of bodyweight. Chinese adapted MMSE (CAMSE) scale was performed to evaluate the cognitive function. According to educational background, different MMSE cut-off values were applied to define dementia: < 24 for participants with ≥ 7 years of education, < 20 for those with 1–6 years of education, and < 17 for illiterate participants. Multivariable linear regression and multivariable binary logistic regression analyses were conducted to assess the associations between WWI and MMSE and dementia, respectively.

**Results:**

Overall, the mean age was 63.7 ± 9.7 years, and 49.0% were males. Multivariate linear regression analyses showed that WWI was negatively associated with MMSE (*β*, -1.09; 95% confidence interval [CI]: -1.24, -0.94). Consistently, multivariable binary logistic regression analyses found a positive association between WWI and the risk of dementia (odds ratio [OR], 1.45; 95% CI: 1.35, 1.56). Compared with individuals in quartile 1 of WWI, the adjusted *β* and OR values of WWI for MMSE and dementia were -2.28 (95% CI: -2.62, -1.94) and 2.12 (95% CI: 1.81, 2.48), respectively. Results of smoothing curve fitting confirmed the linear association between WWI and MMSE and dementia. Subgroup analysis showed a stronger association between WWI and dementia in participants with hypertension with midday napping.

**Conclusion:**

WWI was independently and positively associated with dementia among the population with hypertension, especially in those with midday napping. The data suggests that WWI may serve as a simple and effective tool for the assessment of the risk of dementia in clinical practice.

**Supplementary Information:**

The online version contains supplementary material available at 10.1186/s12888-023-05027-w.

## Introduction

An estimated 4.6 million new cases of dementia have been detected worldwide each year, and the prevalence of dementia will increase rapidly in the coming decades [1]. By 2050, approximately 85 million people are predicted to have dementia in less developed regions. Obesity is widely recognized as a risk factor for various chronic diseases such as cardiovascular disease, metabolic syndrome, stroke, diabetes, and cancer [2–4]. In recent years, the relationship between obesity and dementia has been increasingly reported except in China, but the conclusions are controversial. Some studies found that obesity was associated with cognitive decline [5, 6] and increased the risk of dementia [7, 8]. However, recent findings indicated that obesity failed to increase the risk of dementia [9, 10], while some reported that individuals with obesity have a lower risk for dementia [11, 12]. One major explanation for these inconsistent findings on the obesity-dementia link may be an overreliance on body mass index (BMI) as a measure of obesity [13]. Therefore, further research on obesity should rely less on BMI or at least perform other measurements in addition to BMI.

As a new anthropometric measure reflecting fat and muscle mass components,weight-adjusted waist index (WWI) was calculated by standardizing waist circumference (WC) for bodyweight by combining the advantages of WC and weakening the correlation with BMI [14]. Existing studies have shown a better effect of WWI for predicting the risk of certain diseases (e.g., hypertension and cardiometabolic diseases) than that of WC and BMI, the most commonly used obesity indices. Furthermore, recent findings from our team also found that WWI rather than WC and BMI better predicted the risk of mortality [15]. In other words, WWI may be a comprehensive and effective obesity index.

Therefore, the present study aimed to assess the association between WWI and the risk of dementia and examine any possible effect modifier using data from the China H-type hypertension registry study.

## Methods

### Study participants

The participants in this study was from the China H-type Hypertension Registry Study (registration number: ChiCTR1800017274). The method of data collection and the xclusion criteria in detail have been previously published in prvious literature [16]. Briefly, it is a real-world, observational study, and aimed to determine the prevalence and control situation of hypertension and explore the prognostic factors in China. Eligible participants were male and female individuals aged 18 years and older with hypertension. Exclusion criteria were described as follows: (1) mental or neurological abnormalities that prevent cooperation with the investigation, (2) unable to be followed up for poor adherence or planning to relocate recently, (3) the participants assessed by the study physicians as unsuitable for inclusion or long-term follow-up. The present study was conducted in Wuyuan county in Jiangxi province of China from March 2018 to August 2018 and approved by the Ethics Committee of Anhui Medical University Biomedical Institute (No.CH1059). All participants signed written informed consent.

As a result, a total of 14,234 participants with hypertension were recruited for this study. After excluding participants without Chinese adapted MMSE (CAMSE) data (*n* = 3,945), 10,289 participants were finally included in the present cross-sectional study for analysis.

### Data collection and variable definitions

Demographic information (e.g., age, sex, and education), living conditions (e.g., current smoking, current drinking, midday napping, sleeping, economic level, labor intensity, and psychological stress), dietary habit (e.g., cooking oil, bean products, meat, fruit, and vegetables), self-reported medical history (e.g., diabetes, stroke, coronary heart disease [CHD], chronic renal disease [CKD], and malignant tumor), and medication history (e.g., antihypertensive, glucose-lowering, and lipid-lowering drugs) from all participants were collected using a standard questionnaire by trained staffs.

Current smoking was defined as smoking ≥ 1 cigarette per day for 1 year or more or a cumulative smoking amount ≥ 360 cigarettes per year [17]. Current drinking defined as drinking alcohol an average of at least two or more times a week over a year [17]. Work-time physical activity intensity was defined as mild (light labour), moderate (tired or heavy labour), or severe (extremely tired or heavy labour) according to the self-reported evaluation at work [18]. Economic level was subjectively assessed by participants based on comparision with local residents, which included “good”, “medium”, and “poor” [19]. Psychological stress was referred to the participant's anxiety and emotional tension caused by various reasons such as work problems and health problems, which included “mild”, “modrate”, and “severe” [19]. Sleep duration was assessed by the question: “How many hours on average do you sleep per night?” Mean sleep duration was categorized into 3 groups: ≤ 5, 6–8, and ≥ 9 h [20]. Midday napping was assessed by asking “Did you have midday napping habit?” with the following responses: “yes”, “no” [21].

Physical indicators such as height, weight, and WC were measured twice, and the average values were calculated as the corresponding values. Height was measured using a standard right-angle device and a fixed vertical ruler to the nearest 0.1 cm. Bodyweight was measured using the Omron body fat and weight measurement device from Japan to the nearest 0.01 kg. WC was measured using a tape to measure the minimum circumference at the midpoint between the costal margin and iliac crests (at the umbilicus level) to the nearest 0.1 cm. Blood pressure (BP) was measured three times on the right arm positioned at the heart level using an electronic BP monitor (Omron HBP-1300; OMRON, Japan) after a rest for 5 min, with a 30 s interval between measurements, and three measurements of systolic blood pressure (SBP) and diastolic blood pressure (DBP) were averaged as the corresponding BP values for analysis. BMI was calculated as the weight (kg) divided by the squared height (m^2^). In addition, WWI was calculated as WC (cm) divided by the square root of weight (kg) [14].

Hypertension was defined as seated resting SBP ≥ 140 mmHg or DBP ≥ 90 mmHg or the use of antihypertensive medications at screening [22]. Diabetes mellitus was defined as a self-reported physician diagnosis of diabetes or the use of glucose-lowering drugs or fasting blood glucose (FBG) concentration ≥ 7.0 mmol/L [23]. Dyslipidemia was defined as total cholesterol (TC) ≥ 6.2 mmol/L or triglycerides (TG) ≥ 2.3 mmol/L or low-density lipoprotein cholesterol (LDL-C) ≥ 4.1 mmol/L or high-density lipoprotein cholesterol (HDL-C) < 1.0 mmol/L; or self-reported physician diagnosed dyslipidemia or lipid-lowering drugs use [24].

### Laboratory tests

Blood samples were collected after ≥ 8 h fasting and delivered to a standardized laboratory within 24 h of sampling. Laboratory indicators include plasma homocysteine (Hcy), FBG, TC, TG, HDL-C, LDL-C, uric acid (UA), and creatinine (Cr) were detected from all participants. The estimated glomerular filtration rate (eGFR) was calculated using the Chronic Kidney Disease Epidemiology Collaboration (CKD-EPI) equation [25]. All laboratory measurements followed a standardization and certification program.

### Cognitive assessment

The cognitive function was evaluated using the Chinese version of the MMSE scale [26], which involves a broad series of cognitive domains, including language and visuospatial construction, short-term verbal memory, calculation and attention, immediate recall, and orientation. Correct answers to all questions on the MMSE scale were rated as a maximum score of 30, representing the best cognitive function level. The MMSE score < 24 for participants with secondary school or above education setting (≥ 7 years of education), < 20 for those with primary school (1–6 years of education), and < 17 for illiterate participants were defined as dementia.

### Statistical analysis

Continuous variables were presented as mean ± standard deviation (SD), and categorical variables were expressed as frequency (n) and percentage (%). The characteristic differences by WWI quartiles were contrasted using one-way analysis of variance (ANOVA) tests for continuous variables or Chi-square tests for categorical variables. The associations between WWI and MMSE and dementia were analyzed using multivariable linear regression and multivariable binary logistic regression, respectively. The applicable conditions of regression analysis were checked to be qualified. In addition, the results were described as *β* coefficients and odds ratios (ORs) with 95% confidence intervals (CIs). We conducted three levels of adjustment models for the regression analysis: model 1was adjusted for none; model 2 was adjusted for age and sex; and model 3 was adjusted for age, sex, BMI, SBP, DBP, Hcy, FPG, TC, TG, HDL-C, LDL-C, UA, eGFR, diabetes, stroke, CHD, CKD, malignant tumor, antihypertensive drugs, glucose-lowering drugs, lipid-lowering drugs, current smoking, current drinking, midday napping, sleeping duration, economic level, labor intensity, psychological stress, cooking oil, bean products, meat, fruits, and vegetables. These covariables adjusted in the regression models were selected due to clinical importance, statistical significance in the univariable analysis, and the potential confounder effect estimates individually changed by at least 10%. Dose–response relationship between WWI and MMSE and dementia were evaluated using a generalized additive model (GAM) and a fitted smoothing curve (penalized spline method). Additionally, possible modifications of the association with dementia were evaluated using stratified analyses and interaction testing.

R statistical package (http://www.r-proje.ct.org) and Empower (R) software (www.empow.erstats.com) were used for statistical analyses. Statistical significance was defined as a two-tailed *P* < 0.05.

## Results

### Basic characteristics

The basic characteristics of 10,289 participants grouped by WWI quartiles are shown in Table 1. Overall, the mean (SD) age was 63.7 (9.7) years old, and 49.0% were males. The mean ± SD values of WWI, WC, and BMI were 11.1 ± 0.8 cm/√kg, 84.0 ± 10.0 cm, and 23.6 ± 3.5 kg/m^2^, respectively. Participants with higher WWI were more likely to be older, females, have higher BMI, WC, SBP, DBP, FPG, TC, TG, LDL-C, UA, lower Hcy, HDL-C, eGFR, MMSE score (including MMSE subscores), a higher rate of diabetes, use of antihypertensive and glucose-lowering drugs, a lower proportion of current smokers, drinkers, a weaker labor intensity, and less weekly intake of bean products and meat (all *P* < 0.05).

**Table1:** Baseline characteristics of participants stratified by quartiles of WWI

Characteristics	Total	WWI, cm/*√*kg				
		Q1 (≥ 6.3, <10.6)	Q2 (≥ 10.6, <11.1)	Q3 (≥ 11.1, <11.6)	Q4 (≥ 11.6, <25.8)	*P*-value
N	10,289	2570	2574	2572	2573	
Age, years	63.7 ± 9.7	62.3 ± 10.0	62.3 ± 10.1	63.9 ± 9.2	66.6 ± 9.0	< 0.001
Male, n(%)	5039 (49.0)	1741 (67.7)	1550 (60.2)	1154 (44.9)	594 (23.1)	< 0.001
Physical examination						
BMI, kg/m^2^	23.6 ± 3.5	21.6 ± 3.2	23.4 ± 3.2	24.3 ± 3.2	25.0 ± 3.6	< 0.001
WC, cm	84.0 ± 10.0	75.2 ± 7.9	82.9 ± 7.8	86.6 ± 7.8	91.1 ± 8.8	< 0.001
SBP, mmHg	147.0 ± 17.6	144.8 ± 17.6	146.2 ± 17.4	147.5 ± 17.5	149.4 ± 17.5	< 0.001
DBP, mmHg	88.8 ± 10.8	87.3 ± 10.5	89.0 ± 11.0	89.2 ± 10.5	89.8 ± 11.0	< 0.001
WWI, cm/kg	11.1 ± 0.8	10.1 ± 0.4	10.8 ± 0.1	11.3 ± 0.1	12.1 ± 0.6	< 0.001
Laboratory results						
Hcy (μmol/L)	18.1 ± 11.4	18.9 ± 12.3	18.1 ± 11.3	17.8 ± 11.8	17.4 ± 10.0	< 0.001
FPG (mmol/L)	6.2 ± 1.6	5.9 ± 1.3	6.1 ± 1.5	6.3 ± 1.7	6.4 ± 1.8	< 0.001
TC (mmol/L)	5.1 ± 1.1	5.0 ± 1.1	5.0 ± 1.1	5.2 ± 1.1	5.2 ± 1.1	< 0.001
TG (mmol/L)	1.8 ± 1.3	1.5 ± 1.1	1.8 ± 1.3	2.0 ± 1.3	2.1 ± 1.3	< 0.001
HDL-C (mmol/L)	1.5 ± 0.4	1.6 ± 0.4	1.5 ± 0.4	1.5 ± 0.4	1.5 ± 0.4	< 0.001
LDL-C (mmol/L)	2.9 ± 0.8	2.7 ± 0.7	2.9 ± 0.8	3.0 ± 0.8	3.1 ± 0.8	< 0.001
UA (μmol/L)	429.1 ± 121.0	425.5 ± 119.4	435.1 ± 122.0	428.6 ± 123.9	427.0 ± 118.3	0.024
eGFR ( mL/min/1.73 m^2^)	86.0 ± 19.7	86.2 ± 20.3	87.0 ± 19.7	86.7 ± 18.8	84.0 ± 19.7	< 0.001
Cognitive Function						
Language and visual -spatial skills	7.0 ± 1.7	7.4 ± 1.7	7.3 ± 1.7	6.9 ± 1.7	6.3 ± 1.6	< 0.001
Short-term verbal memory	1.9 ± 1.2	2.0 ± 1.2	2.0 ± 1.2	1.9 ± 1.2	1.7 ± 1.3	< 0.001
Calculation and attention	2.7 ± 2.0	3.1 ± 1.9	3.0 ± 1.9	2.7 ± 1.9	2.1 ± 1.9	< 0.001
Immediate recall	2.3 ± 1.0	2.5 ± 0.9	2.4 ± 1.0	2.3 ± 1.0	2.1 ± 1.1	< 0.001
Orientation	8.1 ± 2.3	8.6 ± 2.0	8.5 ± 2.0	8.0 ± 2.3	7.3 ± 2.5	< 0.001
MMSE	22.0 ± 6.4	23.6 ± 5.8	23.2 ± 6.1	21.8 ± 6.4	19.5 ± 6.5	< 0.001
Dementia	3102 (30.1)	550 (21.4)	623 (24.2)	790 (30.7)	1139 (44.3)	< 0.001
Medical history, n (%)						
Diabetes	1898 (18.4)	306 (11.9)	426 (16.6)	547 (21.3)	619 (24.1)	< 0.001
Dyslipidemia	3672 (35.7)	628 (24.4)	877 (34.1)	1051 (40.9)	1116 (43.4)	< 0.001
Stroke	762 (7.4)	196 (7.6)	196 (7.6)	190 (7.4)	180 (7.0)	0.806
CHD	598 (5.8)	142 (5.5)	138 (5.4)	152 (5.9)	166 (6.5)	0.346
CKD	613 (6.0)	180 (7.0)	141 (5.5)	148 (5.8)	144 (5.6)	0.076
Malignant tumor	126 (1.2)	34 (1.3)	36 (1.4)	32 (1.2)	24 (0.9)	0.445
Medication history, n (%)						
Antihypertensive drugs	6416 (62.4)	1536 (59.8)	1554 (60.4)	1635 (63.6)	1691 (65.7)	< 0.001
Glucose-lowering drugs	513 (5.0)	68 (2.6)	107 (4.2)	159 (6.2)	179 (7.0)	< 0.001
Lipid-lowering drugs	380 (3.7)	74 (2.9)	99 (3.8)	105 (4.1)	102 (4.0)	0.086
Living conditions						
Current smoking, n (%)	2700 (26.2)	918 (35.7)	794 (30.8)	603 (23.4)	385 (15.0)	< 0.001
Current drinking, n (%)	2220 (21.6)	729 (28.4)	678 (26.3)	509 (19.8)	304 (11.8)	< 0.001
Midday napping, n (%)	5503 (53.5)	1401 (54.5)	1399 (54.4)	1359 (52.8)	1344 (52.2)	0.270
Sleeping duration, n (%)						
≤ 5 h	429 (4.2)	109 (4.2)	93 (3.6)	103 (4.0)	124 (4.8)	0.341
6-8 h	5308 (51.6)	1330 (51.8)	1339 (52.0)	1348 (52.4)	1291 (50.2)	
≥ 9 h	4552 (44.2)	1131 (44.0)	1142 (44.4)	1121 (43.6)	1158 (45.0)	
Economic level, n (%)						0.162
Good	1344 (13.1)	317 (12.3)	353 (13.7)	360 (14.0)	314 (12.2)	
Medium	6946 (67.5)	1761 (68.5)	1743 (67.7)	1717 (66.8)	1725 (67.0)	
Poor	1999 (19.4)	492 (19.1)	478 (18.6)	495 (19.2)	534 (20.8)	
Work-time physical activity intensity, n (%)						< 0.001
Mild	5844 (56.8)	1315 (51.2)	1440 (55.9)	1480 (57.5)	1609 (62.5)	
Moderate	2333 (22.7)	645 (25.1)	618 (24.0)	578 (22.5)	492 (19.1)	
Severe	2112 (20.5)	610 (23.7)	516 (20.0)	514 (20.0)	472 (18.3)	
Psychological stress, n (%)						0.099
Mild	6855 (66.6)	1678 (65.3)	1753 (68.1)	1719 (66.8)	1705 (66.3)	
Moderate	2514 (24.4)	630 (24.5)	595 (23.1)	640 (24.9)	649 (25.2)	
Severe	920 (8.9)	262 (10.2)	226 (8.8)	213 (8.3)	219 (8.5)	
Dietary habit						
Cooking oil, n (%)						0.235
Only vegetable oil	1996 (19.4)	481 (18.7)	472 (18.3)	508 (19.8)	535 (20.8)	
Mainly vegetable oil	6815 (66.2)	1724 (67.1)	1738 (67.5)	1685 (65.5)	1668 (64.8)	
Mainly animal oil	1478 (14.4)	365 (14.2)	364 (14.2)	379 (14.7)	370 (14.4)	
Bean products, n (%)						< 0.001
Barely	6853 (66.6)	1635 (63.6)	1608 (62.5)	1740 (67.7)	1870 (72.7)	
1–2 times per week	2529 (24.6)	684 (26.6)	698 (27.1)	624 (24.3)	523 (20.3)	
≥ 3 times per week	907 (8.8)	251 (9.8)	268 (10.4)	208 (8.1)	180 (7.0)	
Meat, n (%)						< 0.001
Barely	2829 (27.5)	644 (25.1)	633 (24.6)	719 (28.0)	833 (32.4)	
1–2 times per week	2855 (27.7)	701 (27.3)	690 (26.8)	695 (27.0)	769 (29.9)	
≥ 3 times per week	4605 (44.8)	1225 (47.6)	1251 (48.6)	1158 (45.0)	971 (37.7)	
Fruits and vegetables, n (%)						0.727
≤ 1.5 kg per week	1664 (16.2)	432 (16.8)	407 (15.8)	403 (15.7)	422 (16.4)	
> 1.5 kg per week	8625 (83.8)	2138 (83.2)	2167 (84.2)	2169 (84.3)	2151 (83.6)	

Data are presented as n (percentage), mean ± standard deviation. *BMI* Body mass index, *WC* Waist circumference, *SBP* Systolic blood pressure, *DBP* Diastolic blood pressure, *WWI* Weight-adjusted waist index, *FPG* Fasting plasma glucose, *TC* Total cholesterol, *TG* Triglyceride, *HDL-C* High-density lipoprotein cholesterol, *LDL-C* Low-density lipoprotein cholesterol, *UA* Uric acid, *eGFR* Estimated glomerular filtration rate, *CHD* Coronary heart disease, *CKD* Chronic renal disease

### Association between WWI, BMI and dementia

**Fig. 1:**
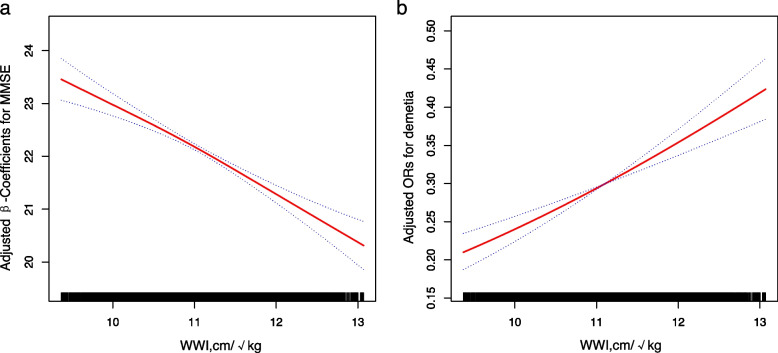
Dose-respone relationship of WWI with MMSE (**a**) and dementia (**b**). Adjusted for age, sex, BMI, SBP, DBP, Hcy, FPG, TC, TG, HDL-C, LDL-C, UA, eGFR, diebetes, stroke, CHD, CKD, malignant tumor, antihypertensive drugs, glucose-lowering drugs, lipid-lowering drugs, current smoking, current drinking, midday napping, sleeping duration, economic level, labour intensity, psychological stress, cooking oil, bean products, meat, fruits and vegetables

Overall, a significant linear association between WWI and MMSE score and the risk of dementia was presented in the smoothing curve (Fig. 1a and b). For 1 unit increment in WWI, the MMSE score was changed by -1.09 (95% CI: -1.24, -0.94) according to the estimation from regression *β* coefficients. In addition, the OR for the risk of dementia was 1.45 (95% CI: 1.35, 1.56).

As shown in Table 2, a negative association between WWI and MMSE score was detected in all models (models 1–3) after adjusting for potential confounders. When WWI was assessed as quartiles, in the final adjusted model (model 3), the adjusted *β* of WWI on MMSE for participants in quartiles 2, 3, and 4 were -0.62 (95% CI: -0.92, -0.33), -1.31 (95% CI: -1.63, -1.00), and -2.28 (95% CI: -2.62, -1.94), respectively, compared with those in quartile 1 (*P* for trend < 0.001). The negative association between WWI and the scores in the five domains of MMSE was presented in Supplementary Table 1. In Table 3, compared with participants in the quartile 1, the adjusted ORs of WWI on dementia for participants in the quartiles 2, 3, and 4 were 1.29 (95% CI: 1.11, 1.49), 1.56 (95% CI: 1.34, 1.81), and 2.12 (95% CI: 1.81, 2.48), respectively. Meanwhile, the *P* for trend in all models was significant, indicating a dose–response relationship between WWI and MMSE score and dementia.

**Table 2:** Association of WWI with MMSE in different models

WWI	MMSE, β(95%CI)		
	Model 1	Model 2	Model 3
Continuous	-2.02 (-2.17, -1.87)	-0.72 (-0.86, -0.57)	-1.09 (-1.24, -0.94)
Quartiles			
Q1 (≥ 6.3, < 10.6)	0.00	0.00	0.00
Q2 (≥ 10.6, < 11.1)	-0.39 (-0.73, -0.06)	-0.04 (-0.34, 0.27)	-0.62 (-0.92, -0.33)
Q3 (≥ 11.1, < 11.6)	-1.81 (-2.15, -1.47)	-0.50 (-0.81, -0.19)	-1.31 (-1.63, -1.00)
Q4 (≥ 11.6, < 25.8)	-4.14 (-4.48, -3.80)	-1.40 (-1.72, -1.07)	-2.28 (-2.62, -1.94)
*P* for trend	< 0.001	< 0.001	< 0.001

Model 1: adjusted none

Model 2: adjusted age, sex

Model 3: adjusted age, sex, BMI,SBP, DBP, Hcy, FPG, TC, TG, HDL-C, LDL-C, UA, eGFR, diebetes, stroke, CHD, CKD, malignant tumor, antihypertensive drugs, glucose-lowering drugs, lipid-lowering drugs, current smoking, current drinking, midday napping, sleeping duration, economic level, work-time physical activity intensity, psychological stress, cooking oil, bean products, meat, fruits and vegetables

**Table 3:** Association of WWI with dementia in different models

WWI	Dementia,OR(95%CI)		
	Model 1	Model 2	Model 3
Continuous	1.76 (1.66, 1.87)	1.25 (1.18, 1.33)	1.45 (1.35, 1.56)
Quartiles			
Q1 (≥ 6.3, < 10.6)	1.00	1.00	1.00
Q2 (≥ 10.6, < 11.1)	1.17 (1.03, 1.34)	1.06 (0.92, 1.22)	1.29 (1.11, 1.49)
Q3 (≥ 11.1, < 11.6)	1.63 (1.44, 1.85)	1.18 (1.03, 1.35)	1.56 (1.34, 1.81)
Q4 (≥ 11.6, < 25.8)	2.92 (2.58, 3.30)	1.53 (1.34, 1.75)	2.12 (1.81, 2.48)
*P* for trend	< 0.001	< 0.001	< 0.001

Model 1: adjusted none

Model 2: adjusted age, sex

Model 3: adjusted age, sex, BMI,SBP, DBP, Hcy, FPG, TC, TG, HDL-C, LDL-C, UA, eGFR, diebetes, stroke, CHD, CKD, malignant tumor, antihypertensive drugs, glucose-lowering drugs, lipid-lowering drugs, current smoking, current drinking, midday napping, sleeping duration, economic level, work-time physical activity intensity, psychological stress, cooking oil, bean products, meat, fruits and vegetables

However, BMI was positively associated with MMSE score (Supplemental Table 2, *β:*0.15, 95%CI:0.11, 0.19), and negatively associated with dementia ( Supplemental Table 3, OR:0.95, 95%CI:0.94, 0.97).

### Subgroup analyses

**Fig. 2:**
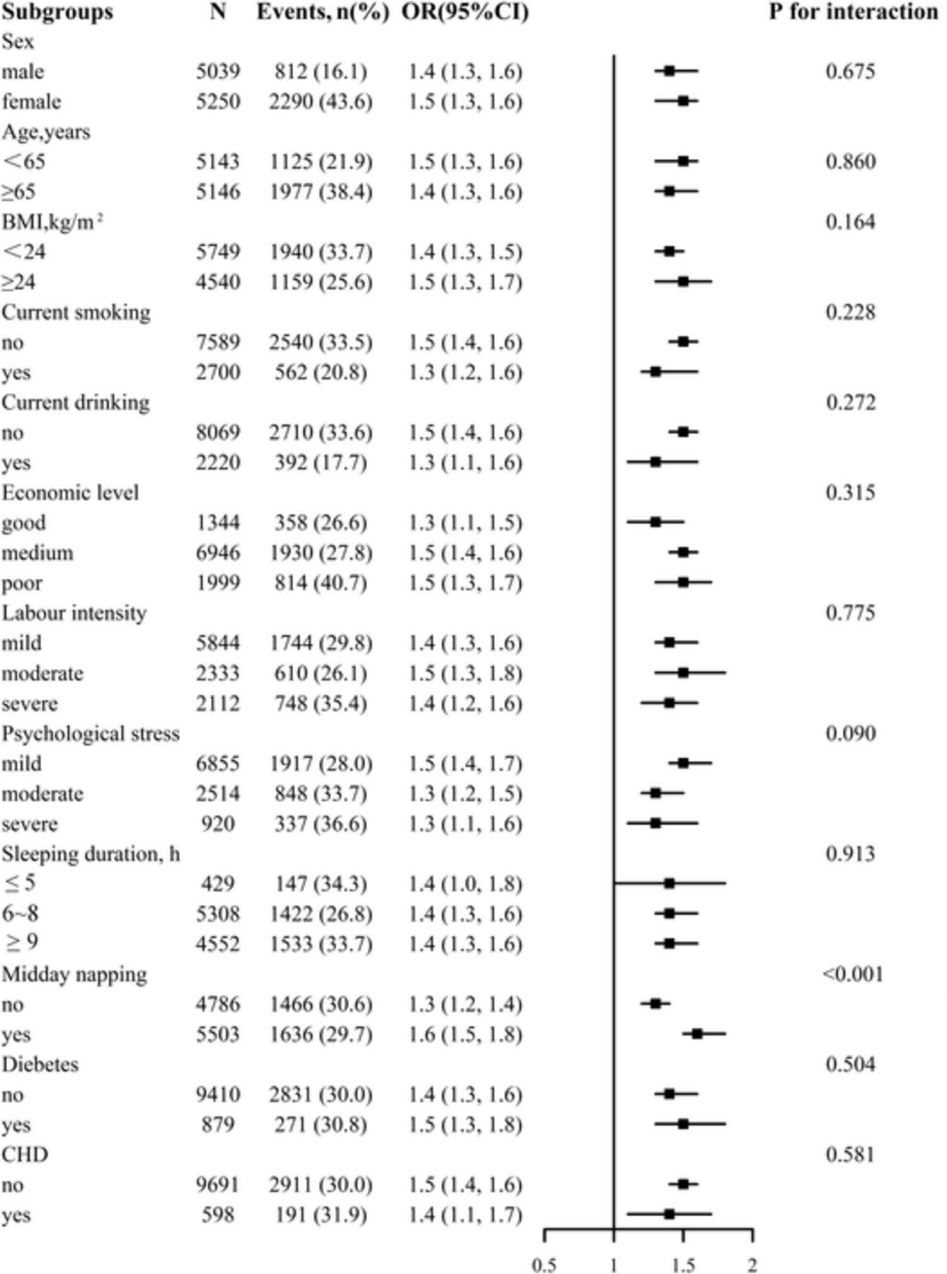
Subgroup analyses of the effect of WWI on dementia. Each subgroup analysis adjusted for age, sex, BMI, SBP, DBP, Hcy, FPG, TC, TG, HDL-C, LDL-C, UA, eGFR, diebetes, stroke, CHD, CKD, malignant tumor, antihypertensive drugs, glucose-lowering drugs, lipid-lowering drugs, current smoking, current drinking, midday napping, sleeping duration, economic level, labour intensity, psychological stress, cooking oil, bean products, meat, fruits and vegetables, except for the stratifying variable. BMI, Body mass index; CHD, coronary heart disease

Stratified analyses were performed to evaluate the effect of WWI (per 1 unit increment) on dementia in various subgroups (Fig. 2). A significant interaction was found in the subgroup of midday napping (*P* for interaction < 0.05), and a stronger association between WWI and dementia was found in participants with midday napping, whereas in the other subgroups, such as sex (male vs. female), age (< 65 vs. ≥ 65 y), BMI (< 24 vs. ≥ 24 kg/m^2^), current smoking (no vs. yes), current drinking (no vs. yes), economic level (good vs. medium vs. poor), labor intensity (mild vs. moderate vs. severe), psychological stress (mild vs. moderate vs. severe), sleeping duration (≤ 5 vs. 6–8 vs. ≥ 9 h), diabetes (no vs. yes), and CHD (no vs. yes), consistent association were observed (all *P* for interaction > 0.05).

## Discussion

This cross-sectional population-based study suggested that WWI, a new obesity index, was independently associated with dementia. There was a linear negative association between WWI and MMSE score and a linear positive association between WWI and dementia. In addition, a stronger association between WWI and dementia was found among participants with midday napping than those without midday napping.

Several previous studies regarding the relationship between obesity and cognitive function showed inconsistent results. A longitudinal study conducted on adults with hypertension reported that a greater degree of adiposity, as measured by BMI, is independently associated with slower cognitive decline [27]. Similarly, we found that BMI was negatively associated with dementia. However, another longitudinal study revealed that obesity defined using BMI in middle age increased the risk of cognitive impairment in old age. Other research showed a null association between BMI and recognition performance [10, 28]. In addition, Luchsinger et al. confirmed that the relationship between BMI and dementia was U-shaped in a prospective study [29]. Those complex and inconsistent results are largely owing to overreliance on BMI as a measure of obesity. BMI is not a suitable measure for reflecting the impact of obesity on cognitive performance in adults. Recently, many researchers used imaging examinations to explore the effects of obesity on cognitive performance. An observational study conducted by Yoon et al. in Korean older adult populations used computed tomography (CT) scanning to measure adipose tissue and demonstrated that higher adiposity was associated with poorer cognitive performance [30]. Additionally, a significant inverse correlation between visceral adipose tissue measured by magnetic resonance imaging (MRI) and cognition has been reported by Isaac et al. [31]. Obesity is represented by excess accumulation of fat in adipose tissue, and imaging examinations are the “gold standard” for the evaluation of adipose tissue. Nevertheless, imaging examinations to assess obesity are often too costly, limiting its extensively clinical application.

Our study used WWI, a new surrogate index of obesity, to explore the association between obesity and dementia. Our findings agreed well with the results obtained by imaging examinations, which quantitatively estimate the fat in adipose tissue to reflect obesity status directly [30, 31]. Most previous works on obesity on cognitive impairment and dementia were limited by overreliance on BMI as an index to define obesity rather than specific body fat and muscle mass measures. Aging is accompanied by increased body fat and reduces lean mass without overall weight loss. However, BMI cannot discriminate between fat and lean mass and has limited value in capturing these age-related changes in older adults [32, 33]. Of note, studies have indicated that reduced lean mass was associated with a higher risk of dementia [34, 35]. WC is a simple and powerful anthropometric index for abdominal fat, which is better at reflecting visceral obesity than that of BMI [36]. A prospective study performed by Cho et al. investigated the association between WC and dementia in Korean older adults, reporting that a significantly higher risk of dementia is associated with abdominal obesity measured by WC [37]. However, although WC can reflect regional fat distribution, it is highly correlated with BMI [38]. WWI, calculated as WC divided by the square root of weight, assesses adiposity by standardizing WC for weight. Thus, it may combine the advantages of WC and attenuate the association with BMI. Studies have shown that WWI is highly related to all-cause and cardiovascular mortality, hypertension, and metabolic status [15, 16, 39, 40]. Kim et al. used bioelectrical impedance analysis, dual-energy X-ray absorptiometry, and abdominal computed tomography to measure body composition, confirming that WWI was negatively correlated with muscle mass and positively correlated with adipose tissue in Korean older adults [41]. Further study indicated that WWI could assess muscle and fat mass in multi-ethnic populations [42]. Therefore, WWI is a suitable anthropometric index to investigate the impact of obesity on dementia.

The present finding that obesity impacts cognitive outcomes may be involved multiple potential mechanisms. One of the mechanisms may be that excess adipose tissue, as an endocrine organ, releases large amounts of inflammatory cytokines, including interleukin-6 and tumor necrosis factor-α [43, 44]. Moreover, interleukin-6 stimulates the release of C-reactive protein from the liver [45]. Such inflammatory factors have been demonstrated to exert a detrimental impact on cognitive performance [46, 47]. Another plausible mechanism is involved in obesity-linked leptin resistance. Leptin, a hormone produced by adipose tissue, was reported to exert beneficial effects on cognitive function [48]. However, adipose tissue expansion leads to a higher level of circulating leptin, resulting in a high level of leptin resistance [49]. Furthermore, other possible mechanisms were proposed, including impaired cerebral metabolism [50], vascular endothelial damage [51], insulin resistance [52], and neuronal degradation [53].

The implication of midday napping on the dementia was inconsistently reported. A longitudinal study conducted in cognitively unimpaired older individuals found that daytime napping was independently associated with slower cognitive decline [54]. Another cohort study indicated the cross-sectional association between longer napping duration and poorer cognitive function in the elderly [55]. One of crucial reasons for these differences could be due to neglect of the nighttime sleep quality and duration. In the present study, we found that there was a stronger association between WWI and dementia among participants with midday napping, and they experienced longer sleep duration and better sleep quality. A recent publication by Leng et al. suggested that people with longer sleep duration and higher sleep efficiency have a greater risk of cognitive impairment with midday napping [56], which was consistent with our findings. Midday napping may affect cognition indirectly through total sleep duration [57, 58]. Finally, long-term midday napping might result in a sedentary lifestyle and impaired social communication, both of which were related to cognitive decline.

Our study has several strengths, including the large sample size study design. To our knowledge, this is the first time to evaluate the potential relationship between WWI and dementia in China. In our analysis, we explore the dose–response association between WWI and dementia using GAM and penalized spline method. An advantage over previous studies on factors affecting dementia is that our study considered the effects of some new covariates, including sleeping, midday napping, labor intensity, psychological stress, and diet on dementia. We focused on the impact of life behaviors on dementia, and we found that WWI was more strongly associated with dementia in participants with midday napping than those without midday napping.

Nevertheless, several limitations should be considered. First, our participants were Chinese people with hypertension, which would limit the generalizability of our findings to other populations. The casual relationship between WWI and dementia will be elucidated by the further prospective cohort studies rather than the present cross-sectional study. Second, the detailed information about midday napping duration and efficiency was not collected, so we could not analyze the specific effect of napping on the association between WWI and dementia. Third, although we analysed the data basing on a large sample, more than a quarter of total participants randomly missed data of MMSE. Fourth, there was a limitation to define dementia solely on the basis of MMSE. Finally, although various covariates were adjusted, residual confounding may still be possible.

## Conclusions

In summary, we found a linear positive association between WWI and dementia in the Chinese population with hypertension. The data suggest that WWI may serve as a simple and effective tool for the assessment of the risk of dementia in daily clinical practice.

## Supplementary Information

**Additional file 1: Table 1. **Association of WWI with scores in five domains of MMSE in different models. **Table 2. **Association between BMI and MMSE in different models. **Table 3.** Association between BMI and dementia in different models. **Table A. **Characteristics of participants included and excluded.** Table B.** Collinearity test results for continuous covariates. **Table C. **ROC analysis on BMI, WC and WWI assessing the risk of dementia. **Table D. **Characteristics of participants with midday napping and without midday napping. 

## Data Availability

The datasets analyzed in the present study will be available from the corresponding author upon reasonable request.

## Abbreviations

WWI: Weight-adjusted waist index
WC: Waist circumference
MMSE: Chinese adapted MMSE (CAMSE)
BMI: Body mass index
CHD: Coronary heart disease
CKD: Chronic renal disease
BP: Blood pressure
SBP: Systolic blood pressure
DBP: Diastolic blood pressure
FBG: Fasting blood glucose
Hcy: Homocysteine
TC: Total cholesterol
TG: Triglycerides
HDL-C: High-density lipoprotein cholesterol
LDL-C: Low-density lipoprotein cholesterol
UA: Uric acid
Cr: Creatinine
eGFR: Estimated glomerular filtration rate
CKD-EPI: Chronic Kidney Disease Epidemiology Collaboration
CT: Computed tomography
MRI: Magnetic resonance imaging
SD: Standard deviation
CI: Confidence interval
OR: Odds ratio
ANOVA: One-way analysis of variance
GAM: Generalized additive model
