# Supplementary material for: Positive association between weight-adjusted-waist index and dementia in the Chinese population with hypertension: a cross-sectional study
Source: BMC Psychiatry. 2023 Jul 19;23:519. doi: 10.1186/s12888-023-05027-w (PMC10357774; doi:10.1186/s12888-023-05027-w)
Supplement: Supplementary file 1 — Additional file 1: Table 1. Association of WWI with scores in five domains of MMSE in different models. Table 2. Association between BMI and MMSE in different models. Table 3. Association between BMI and dementia in different models. Table A. Characteristics of participants included and excluded. Table B. Collinearity test results for continuous covariates. Table C. ROC analysis on BMI, WC and WWI assessing the risk of dementia. Table D. Characteristics of participants with midday napping and without midday napping. [file 12888_2023_5027_MOESM1_ESM.doc]

Supplementary Table 1. Association of WWI with scores in five domains of MMSE in different models

| WWI | *β* (95%CI) | | |
| --- | --- | --- | --- |
|  | Model 1 | Model 2 | Model 3 |
| Orientation |  |  |  |
| Continuous | -0.69 (-0.74, -0.64) | -0.26 (-0.31, -0.21) | -0.35 (-0.41, -0.30) |
| Quartiles |  |  |  |
| Q1 (≥6.3, ＜10.6) | 0 | 0 | 0 |
| Q2 (≥10.6, ＜11.1) | -0.13 (-0.25, -0.01) | -0.00 (-0.11, 0.11) | -0.15 (-0.26, -0.04) |
| Q3 (≥11.1, ＜11.6) | -0.63 (-0.75, -0.51) | -0.19 (-0.30, -0.07) | -0.39 (-0.50, -0.27) |
| Q4 (≥11.6, ＜25.8) | -1.38 (-1.50, -1.26) | -0.46 (-0.58, -0.34) | -0.68 (-0.80, -0.55) |
| *P* for trend | <0.001 | <0.001 | <0.001 |
| Immediate recall |  |  |  |
| Continuous | -0.17 (-0.19, -0.14) | -0.07 (-0.09, -0.04) | -0.11 (-0.14, -0.08) |
| Quartiles |  |  |  |
| Q1 (≥6.3, ＜10.6) | 0 | 0 | 0 |
| Q2 (≥10.6, ＜11.1) | -0.05 (-0.10, 0.01) | -0.03 (-0.08, 0.03) | -0.09 (-0.15, -0.04) |
| Q3 (≥11.1, ＜11.6) | -0.12 (-0.18, -0.07) | -0.03 (-0.09, 0.02) | -0.13 (-0.18, -0.07) |
| Q4 (≥11.6, ＜25.8) | -0.35 (-0.41, -0.29) | -0.15 (-0.20, -0.09) | -0.25 (-0.31, -0.18) |
| *P* for trend | <0.001 | <0.001 | <0.001 |
| Calculation and attention |  |  |  |
| Continuous | -0.51 (-0.55, -0.46) | -0.16 (-0.21, -0.12) | -0.26 (-0.31, -0.22) |
| Quartiles |  |  |  |
| Q1 (≥6.3, ＜10.6) | 0 | 0 | 0 |
| Q2 (≥10.6, ＜11.1) | -0.14 (-0.25, -0.04) | -0.04 (-0.14, 0.05) | -0.20 (-0.29, -0.10) |
| Q3 (≥11.1, ＜11.6) | -0.46 (-0.57, -0.36) | -0.11 (-0.21, -0.01) | -0.33 (-0.43, -0.23) |
| Q4 (≥11.6, ＜25.8) | -1.05 (-1.16, -0.95) | -0.32 (-0.43, -0.22) | -0.57 (-0.68, -0.46) |
| *P* for trend | <0.001 | <0.001 | <0.001 |
| Short-term verbal memory |  |  |  |
| Continuous | -0.12 (-0.15, -0.09) | -0.03 (-0.07, -0.00) | -0.08 (-0.11, -0.04) |
| Quartiles |  |  |  |
| Q1 (≥6.3, ＜10.6) | 0 | 0 | 0 |
| Q2 (≥10.6, ＜11.1) | -0.01 (-0.07, 0.06) | 0.01 (-0.06, 0.07) | -0.07 (-0.14, -0.00) |
| Q3 (≥11.1, ＜11.6) | -0.08 (-0.15, -0.01) | -0.00 (-0.07, 0.06) | -0.10 (-0.17, -0.03) |
| Q4 (≥11.6, ＜25.8) | -0.26 (-0.32, -0.19) | -0.08 (-0.15, -0.01) | -0.18 (-0.26, -0.10) |
| *P* for trend | <0.001 | 0.044 | <0.001 |
| Language and visual-spatial skills |  |  |  |
| Continuous | -0.53 (-0.57, -0.49) | -0.19 (-0.23, -0.15) | -0.28 (-0.33, -0.24) |
| Quartiles |  |  |  |
| Q1 (≥6.3, ＜10.6) | 0 | 0 | 0 |
| Q2 (≥10.6, ＜11.1) | -0.07 (-0.16, 0.03) | 0.03 (-0.05, 0.11) | -0.12 (-0.20, -0.03) |
| Q3 (≥11.1, ＜11.6) | -0.51 (-0.60, -0.42) | -0.17 (-0.25, -0.08) | -0.37 (-0.45, -0.28) |
| Q4 (≥11.6, ＜25.8) | -1.10 (-1.19, -1.01) | -0.39 (-0.48, -0.30) | -0.61 (-0.70, -0.51) |
| *P* for trend | <0.001 | <0.001 | <0.001 |

Model 1: adjusted none

Model 2:adjusted age, sex

Model 3:adjusted age, sex, SBP, DBP, Hcy, FPG, TC, TG, HDL-C, LDL-C, UA , eGFR, diebetes, stroke, CHD, CKD, malignant tumor, antihypertensive drugs, glucose-lowering drugs, lipid-lowering drugs, current smoking, current drinking, midday napping, sleeping duration, economic leve, [labour](javascript:;) [intensity](javascript:;), psychological stress, cooking oil, bean products, meat, fruits and vegetables.

Supplemental Table 2. Association between BMI and MMSE in different models

| WWI | MMSE, β(95%CI) | | |
| --- | --- | --- | --- |
|  | Model 1 | Model 2 | Model 3 |
| Continuous | 0.24 (0.21, 0.28) | 0.19 (0.16, 0.22) | 0.15 (0.11, 0.19) |
| Quartiles |  |  |  |
| Q1 (≥6.3, ＜10.6) | 0.00 | 0.00 | 0.00 |
| Q2 (≥10.6, ＜11.1) | 1.70 (1.20, 2.21) | 1.28 (0.83, 1.72) | 1.09 (0.66, 1.52) |
| Q3 (≥11.1, ＜11.6) | 2.87 (2.36, 3.39) | 2.12 (1.66, 2.59) | 1.69 (1.22, 2.17) |
| Q4 (≥11.6, ＜25.8) | 3.24 (2.63, 3.85) | 2.40 (1.85, 2.95) | 1.90 (1.34, 2.47) |
| *P* for trend | <0.001 | <0.001 | <0.001 |

Model 1: adjusted none

Model 2:adjusted age, sex

Model 3:adjusted age, sex,WWI,SBP, DBP, Hcy, FPG, TC, TG, HDL-C, LDL-C, UA , eGFR, diebetes, stroke, CHD, CKD, malignant tumor, antihypertensive drugs, glucose-lowering drugs, lipid-lowering drugs, current smoking, current drinking, midday napping, sleeping duration, economic level, [labour](javascript:;) [intensity](javascript:;), psychological stress, cooking oil, bean products, meat, fruits and vegetables.

Supplemental Table 3. Association between BMI and dementia in different models

| WWI | Dementia,OR(95%CI) | | |
| --- | --- | --- | --- |
|  | Model 1 | Model 2 | Model 3 |
| Continuous | 0.94 (0.92, 0.95) | 0.95 (0.93, 0.96) | 0.95 (0.94, 0.97) |
| Quartiles |  |  |  |
| Q1 (≥6.3, ＜10.6) | 1.00 | 1.00 | 1.00 |
| Q2 (≥10.6, ＜11.1) | 0.67 (0.57, 0.79) | 0.72 (0.61, 0.86) | 0.75 (0.62, 0.91) |
| Q3 (≥11.1, ＜11.6) | 0.48 (0.41, 0.57) | 0.55 (0.46, 0.66) | 0.59 (0.48, 0.73) |
| Q4 (≥11.6, ＜25.8) | 0.45 (0.37, 0.56) | 0.53 (0.43, 0.67) | 0.59 (0.45, 0.76) |
| *P* for trend | <0.001 | <0.001 | <0.001 |

Model 1: adjusted none

Model 2:adjusted age, sex

Model 3:adjusted age, sex,WWI,SBP, DBP, Hcy, FPG, TC, TG, HDL-C, LDL-C, UA , eGFR, diebetes, stroke, CHD, CKD, malignant tumor, antihypertensive drugs, glucose-lowering drugs, lipid-lowering drugs, current smoking, current drinking, midday napping, sleeping duration, economic level, [labour](javascript:;) [intensity](javascript:;), psychological stress, cooking oil, bean products, meat, fruits and vegetables.

A total of 14,234 participants with hypertension were recruited for the study. 10289 participants were included in the present analysis, while 3945 participants were excluded for missing mini-mental state examination (MMSE) data. Table A showed that there were no significant differences for basic characteristics between participants included and excluded (all P＞0.05), which means that the missing data in our study is missing at random. Therefore, we may believe that this situation of random missing data will have little impact on our analysis results.

**Table A. Characteristics of participants included and excluded**

| Characteristics | Participants included | Participants excluded | *P*-value |
| --- | --- | --- | --- |
| N | 10289 | 3945 |  |
| Age, years | 63.9±8.3 | 63.7±9.7 | 0.168 |
| WC, cm | 83.5±9.5 | 83.8±9.9 | 0.102 |
| Weight, kg | 57.6±10.8 | 57.7±10.7 | 0.391 |
| WWI, cm/kg | 11.09±0.80 | 11.11±0.82 | 0.190 |
| Male, n(%) | 5039(49.0) | 1902(48.2) | 0.416 |
| Current smoking, n (%) | 2700(26.2) | 993(25.2) | 0.067 |
| Current drinking, n (%) | 2220(21.6) | 848(21.5) | 0.945 |

Table B showed the multicollinearity for continuous covariables adjusted in the regression analysis, and all values of variance inflation factor (VIF ) were less than 5.

Table B Collinearity test results for continuous covariates

| Continuous covariables | VIF values |
| --- | --- |
| WWI | 1.3 |
| BMI | 1.4 |
| SBP | 1.5 |
| DBP | 1.6 |
| Hcy | 1.1 |
| Glu | 1.1 |
| UA | 1.3 |
| eGFR | 1.4 |
| HDL-C | 1.3 |
| LDL-C | 1.2 |
| TC | 1.8 |
| TG | 1.4 |

Table C showed that the AUC values of WC +WWI and BMI+ WWI were slightly higher than the AUC value of WWI, while the AUC value of WWI was higher than that of WC, BMI, and WC+BMI ( all *P* ＜0.05). Therefore, WWI may be a simple and effective indicator for predicting the risk of dementia.

**Table C. ROC analysis on BMI, WC and WWI assessing the risk of dementia**

|  | AUC | 95%CI | Specificity | Sensitivity |
| --- | --- | --- | --- | --- |
| WWI | 0.622 | 0.609, 0.634 | 0.707 | 0.589 |
| WC | 0.566^*^ | 0.554, 0.578 | 0.553 | 0.5496 |
| BMI | 0.569^*^ | 0.557, 0.581 | 0.592 | 0.523 |
| WC+BMI | 0.569^*^ | 0.558, 0.582 | 0.576 | 0.538 |
| WC+WWI | 0.716^*^ | 0.706, 0.727 | 0.629 | 0.686 |
| BMI+WWI | 0.670^*^ | 0.659, 0.682 | 0.696 | 0.559 |

*P＜0.05, compared with WWI

Table D that participants with midday napping experienced longer sleep duration and better sleep quality.

Table D. Characteristics of participants with midday napping and without midday napping

| Characteristics | Participants **without** midday napping | Participants **with**  midday napping | *P*-value |
| --- | --- | --- | --- |
| N | 4786 | 5503 |  |
| Age, years | 63.6 ± 9.8 | 63.9 ± 9.6 | 0.112 |
| SBP, mmHg | 147.3 ± 17.5 | 146.7 ± 17.7 | 0.106 |
| DBP, mmHg | 88.9 ± 10.847 | 88.665 ± 10.732 | 0.155 |
| WWI, cm/kg | 11.110 ± 0.804 | 11.085 ± 0.789 | 0.097 |
| Female, n(%) | 2742 (57.3%) | 2508 (45.6%) | ＜0.001 |
| Medical history, n (%) |  |  |  |
| Stroke | 326 (6.8%) | 436 (7.9%) | 0.062 |
| CHD | 283 (5.9%) | 315 (5.7%) | 0.683 |
| CKD | 280 (5.8%) | 333 (6.1%) | 0.668 |
| Sleeping duration, n (%) |  |  | ＜0.001 |
| ＜5h | 293 (6.1%) | 136 (2.%) |  |
| 5-8h | 3011 (62.9%) | 2297 (41.%) |  |
| ＞8h | 1482 (30.9%) | **3070 (55.8%)** |  |
| Self-report sleeping qualtiy, n (%) |  |  | ＜0.001 |
| well | 2533 (52.9%) | **3455 (62.8%)** |  |
| medium | 1045 (21.8%) | 1024 (18.6%) |  |
| poor | 1208 (25.2%) | 1024 (18.6%) |  |
